# Cytokine Release Syndrome in Cancer Patients Receiving Immune Checkpoint Inhibitors: A Case Series of 25 Patients and Review of the Literature

**DOI:** 10.3389/fimmu.2022.807050

**Published:** 2022-01-28

**Authors:** Sen Hee Tay, Michelle Min Xuan Toh, Yee Liang Thian, Balamurugan A. Vellayappan, Anna-Marie Fairhurst, Yiong Huak Chan, Folefac Aminkeng, Lavina D. Bharwani, Yiqing Huang, Anselm Mak, Alvin Seng Cheong Wong

**Affiliations:** ^1^ Division of Rheumatology, Department of Medicine, National University Hospital, Singapore, Singapore; ^2^ Department of Medicine, Yong Loo Lin School of Medicine, National University of Singapore, Singapore, Singapore; ^3^ Division of Body Imaging, Department of Diagnostic Imaging, National University Hospital, Singapore, Singapore; ^4^ Department of Diagnostic Radiology, Yong Loo Lin School of Medicine, National University of Singapore, Singapore, Singapore; ^5^ Department of Radiation Oncology, National University Cancer Institute, National University Hospital, Singapore, Singapore; ^6^ Singapore Immunology Network, Agency for Science, Technology and Research, Singapore, Singapore; ^7^ Biostatistics Unit, Yong Loo Lin School of Medicine, National University of Singapore, Singapore, Singapore; ^8^ Department of Medical Oncology, Tan Tock Seng Hospital, Singapore, Singapore; ^9^ Department of Haematology-Oncology, National University Cancer Institute, National University Hospital, Singapore, Singapore

**Keywords:** immunotherapy, immune-related adverse events, cytokine release syndrome, tocilizumab, immune checkpoint inhibitors

## Abstract

Cytokine release syndrome (CRS) is a phenomenon of immune hyperactivation described in the setting of immunotherapy. Unlike other immune-related adverse events, CRS triggered by immune checkpoint inhibitors (ICIs) is not well described. The clinical characteristics and course of 25 patients with ICI-induced CRS from 2 tertiary hospitals were abstracted retrospectively from the medical records and analyzed. CRS events were confirmed by 2 independent reviewers and graded using the Lee et al. scale. The median duration of CRS was 15.0 days (Q1; Q3 6.3; 29.8) and 10 (40.0%) had multiple episodes of CRS flares. Comparing the clinical factors and biomarkers in Grades 1-2 and 3-5 CRS, we found that patients with Grades 3-5 CRS had following: (i) had longer time to fever onset [25.0 days (Q1; Q3 13.0; 136.5) vs. 3.0 days (Q1; Q3 0.0; 18.0), p=0.027]; (ii) more cardiovascular (p=0.002), neurologic (p=0.001), pulmonary (p=0.044) and rheumatic (p=0.037) involvement; (iii) lower platelet count (p=0.041) and higher urea (p=0.041) at presentation compared to patients with Grades 1-2 CRS. 7 patients (28.0%) with Grades 1-2 CRS were rechallenged using ICIs without event. 9 patients (36.0%) were treated with pulse methylprednisolone and 6 patients (24.0%) were treated with tocilizumab. Despite this, 3 patients (50%) who received tocilizumab had fatal (Grade 5) outcomes from ICI-induced CRS. Longer time to fever onset, lower platelet count and higher urea at presentation were associated with Grade 3-5 CRS. These parameters may be used to predict which patients are likely to develop severe CRS.

## Introduction

Immune checkpoints, also referred to as inhibitory immune receptors, are well-established negative regulators of the immune response (1). They are essential in maintaining self-tolerance and preventing autoimmunity, and they prevent tissue damage from persistent inflammation (2, 3). Antibodies blocking these immune checkpoints enhance the immune response, with beneficial anti-tumor effects (2). Monoclonal antibodies (mAbs) against 3 of these immune checkpoints; cytotoxic T-lymphocyte-associated antigen 4 (CTLA-4) (e.g. ipilimumab), programmed cell death protein 1 (PD-1) (e.g. pembrolizumab and nivolumab) and its ligand PD-L1 (e.g. atezolizumab, durvalumab and avelumab) are now standard and effective anticancer agents serving as the backbone of cancer therapy for a wide range of cancer types (1, 4–6). However, the same enhancement of the immune response can give rise to undesirable, off-target and inflammatory immune-related adverse events (irAEs) (2, 7). A range of irAEs have been described to occur in almost every organ system, ranging from mild to high grade irAEs that can be fatal (8). Such irAEs are usually managed *via* a multidisciplinary approach by subspecialists with expertise in irAE management (9).

Cytokine release syndrome (CRS) is a phenomenon of immune hyperactivation typically described in the context of chimeric antigen receptor modified T cell (CAR-T) therapy and bispecific T cell receptor engaging (BiTE) immunotherapy (10). Unlike aforementioned irAEs, CRS is a unique, expected, on-target on-tumor toxicity directly related to the mechanisms of cellular and BiTE immunotherapy (11, 12). CRS-induced by immune checkpoint inhibitors (ICIs) has been rarely described in case reports, a single case series and an analysis of the World Health Organization pharmacovigilance database (10, 13–15). However, these reports were unable to comprehensively characterize this toxic event. As such, ICI-induced CRS remains enigmatic and the factors associated with development of high grade ICI-induced CRS have not been characterized. The increasing use of ICIs earlier in treatment paradigms, across a wide range of cancer types, as well as in combination with chemotherapy, targeted therapy and radiotherapy (RT) underscore the need to recognize ICI-induced CRS for prompt evaluation and management.

Although hyperinflammatory syndromes have been recognized in a variety of conditions such as infections and rheumatic disorders, its nosology is still nascent and mostly poorly defined (16). The only exception is the availability of the HScore to diagnose primary and secondary forms of hemophagocytic syndrome (17). In terms of drug-induced hyperinflammation syndrome, Pichler first proposed a classification for adverse effects of biological agents in 2006 and CRS belonged to the Type α reactions, rather than being in the same category with classical hypersensitivity reactions (18). More recently, allergists-immunologists have proposed a new classification and cytokine release is described as a hypersensitivity reaction not responding to premedication or slower infusion rate during the first infusion (19). In the context of immunotherapy, “cytokine storm” has been described in patients receiving mAbs targeting CD3 (e.g. OKT3) or CD28 (e.g. TGN1412) (11, 20). In contrast to cytokine storm, the term CRS in oncology is used to describe the spectrum of reactions seen after the administration of targeted therapies that cause activation of T cells as they recognize tumor antigen (11).

In this article, we report a series of 25 patients evaluated from Feb 2014 to Jan 2021 with CRS that occurred after the administration of ICIs. We report the clinical characteristics of these patients, factors and biomarkers associated with high grade ICI-induced CRS, and their outcomes.

## Materials and Methods

### Patients

We collected data of ICI-induced CRS patients aged 21 years or older treated with at least a single dose of anti-PD-1/PD-L1/CTLA-4/LAG-3-based ICI at National University Hospital, Singapore and Tan Tock Seng Hospital, Singapore from Feb 2014 to Jan 2021. The study was approved by NHG Domain Specific Review Board B (reference code: 2017/01254) and was carried out in accordance with the principles of the Declaration of Helsinki. Subjects gave written informed consent prior to study inclusion. In addition, waiver of consent for retrospective data review from deceased patients was obtained.

### Data Collection

Data including demographic, body mass index, cancer type, specific ICI treatment, personal history of autoimmunity and tumor response were collected retrospectively. Treatment data on the duration, number of cycles of ICI and other treatment modalities, such as concomitant chemotherapy, tyrosine kinase inhibitor (TKI), RT and proton pump inhibitor (PPI) use during ICI were also collected. All patients had systemic inflammatory response syndrome (SIRS) at presentation, meeting 2 or more of the SIRS criteria (21). Two decision rules of different stringency (models A and B) were designed to attribute SIRS events to CRS using attribution Models A and B (**Supplemental Table 1**) (21). We only considered positive microbiological results within 1 week of inpatient investigations for pyrexia, similar to that of pyrexia of unknown origin workup (22). For Model A, all SIRS events without positive microbiological culture results could be attributed to CRS. For Model B, patients could have associated positive microbiological culture results but SIRS events were either attributed to CRS by an infectious disease physician or culture results were deemed to be a contaminant. All rheumatic irAEs were referred to rheumatologists (S.H.T. and A.M.) as part of an immune-related toxicity team referral workflow whereby patients suspected of having irAEs were referred to relevant subspecialists with expertise in their management (9). All patients with suspected ICI-induced CRS were seen by a rheumatologist (S.H.T.) and all data were checked by a medical oncologist (A.S.C.W). Both investigators (S.H.T. and A.S.C.W) resolved any differences in the interpretation and only cases diagnosed to have ICI-induced CRS were included in this study. Severity of CRS events were defined using the Lee et al. grading scale and other irAEs were graded using the Common Terminology Criteria for Adverse Events v5.0 (23). Start of CRS was defined as the day of first fever relative to infusion of ICI and a fever was defined as 38.0°C or higher (24). Resolution of CRS was clinically assessed and documented. Data was collected for patients with multiple episodes of CRS events, if any. For patients with multiple episodes of CRS flares, highest grade of CRS flare was recorded and duration of CRS was reported cumulatively. Non-CRS irAEs were defined in accordance with the guidelines from American Society of Clinical Oncology, European Society for Medical Oncology, National Comprehensive Cancer Network and Society for Immunotherapy of Cancer, while CRS-related toxicities by organ systems were abstracted based on the publication by Brudno et al. (25–29). High-dose vasopressor use was defined by Lee et al. (23). Blood counts data at CRS diagnosis were used to calculate neutrophil-to-lymphocyte ratio (NLR) (absolute neutrophil count/absolute lymphocyte count) and platelet-to-lymphocyte ratio (PLR) (platelet count/absolute lymphocyte count). Cancer treatment responses were defined by Response Evaluation Criteria In Solid Tumors 1.1 criteria as read by a radiologist (Y.L.T.) on serial computed tomography imaging.

### Statistical Analysis

Continuous and categorical data were analyzed using the Mann-Whitney U test and Pearson’s chi-square test, respectively. Kruskal-Wallis test was used for three or more comparisons. Statistical significance was defined as a two-tailed p value of < 0.05. We used the Benjamini-Hochberg (BH) procedure to control the false discovery rate (FDR) and to avoid Type 1 errors from multiple comparisons (30). The FDR is the expected ratio of the number of false positive classifications (false discoveries) to the total number of positive classifications (rejections of the null). FDR adjustments give critical cut-off values for which variables remain statistically significant if the original p values are less than that of the critical values. Herein, we have displayed the FDRs for the readers to appreciate the confidence of the significant p values. All statistical analyses were performed with SPSS, version 27 (IBM Corp, Armonk, NY, US).

## Results

### Patient Characteristics

From Feb 2014 to Jan 2021, 539 patients received ICIs at National University Hospital and Tan Tock Seng Hospital. 25 patients (4.6%) developed symptoms and were diagnosed to have ICI-induced CRS, of which 24 patients (96/0%) and 25 patients (100.0%) fulfilled Models A and B in terms of attribution, respectively. **Table 1** and **Supplemental Table 2** show the characteristics of patients who experienced ICI-induced CRS. The baseline characteristics of the patients who received ICIs from National University Hospital are presented in **Supplemental Table 3**. The median age at diagnosis of CRS was 64.0 years (Q1; Q3 55.0; 74.5), majority of patients had stage IV cancer (96.0%) and were predominantly males (72.0%), in keeping with the data from the larger cohort (**Table 1** and **Supplemental Table 3**). The primary malignancies in this case series were non-small cell lung carcinoma (28.0%), renal cell carcinoma (16.0%), hepatocellular carcinoma (16.0%), melanoma (8.0%) and others. Of note, only 1 patient (4.0%) had pre-existing autoimmune disease, in the form of chronic glomerulonephritis. Monotherapy with anti-PD-1 and anti-PD-L1 was used in 20 patients (80.0%) whereas dual checkpoint blockade with anti-CTLA-4 plus anti-PD-1 was used in 4 patients (16.0%). Some patients had combination treatment with other modalities, which included chemotherapy (24.0%), RT (16.0%) and TKI (16.0%). 88.0% of the patients were receiving concomitant PPIs. None of the patients were on baseline immunosuppressive medications.

**Table 1:** Clinical characteristics in patients with ICI-induced CRS.

Characteristics	Value in patients (n = 25)
Age (years)	64.0 (55.0-74.5)
Gender (male)	18 (72.0)
BMI	22.8 (18.0-25.8)
Ethnicity	
Chinese	17 (68.0)
Malay	4 (16.0)
Indian	3 (12.0)
Others	1 (4.0)
Cancer stage	
IV	24 (96.0)
Cancer type*	
Non-small cell lung cancer	7 (28.0)
Renal cell carcinoma	4 (16.0)
Hepatocellular carcinoma	4 (16.0)
Melanoma	2 (8.0)
Cumulative duration of ICI treatment (days)**	59.5 (21.0-136.5)
Class of ICI treatment	
Anti-PD-1	17 (68.0)
Anti-PD-L1	3 (12.0)
Anti-CTLA-4/Anti-PD-1	4 (16.0)
Anti-LAG-3/Anti-PD-1	1 (4.0)
Cumulative dose of ICI**	
Pembrolizumab (mg)	250.0 (200.0-950.0)
Nivolumab (mg)	800.0 (340.0-1340.0)
Ipilimumab (mg)	107.0 (53.5-187.5)
Cumulative number of cycles**	
Pembrolizumab	2.5 (1.0-4.8)
Nivolumab	5.0 (3.0-9.5)
PD-L1 tumor proportion score (%)	1.0 (0.0-85.0)
Concomitant chemotherapy	6 (24.0)
Concomitant RT	4 (16.0)
Concomitant TKI	4 (16.0)
Concomitant PPI	22 (88.0)
Pre-existing autoimmune disease	1 (4.0)

*Other cancer types included adrenocortical carcinoma, breast cancer, colorectal cancer, endometrial carcinoma, esophageal cancer, nasopharyngeal carcinoma, small cell lung cancer and transitional cell carcinoma (n = 1 for each cancer type).

**Inclusive of data from patients who were rechallenged to ICI.

BMI, body mass index; RT, radiotherapy; TKI, tyrosine kinase inhibitor; PPI, proton pump inhibitor. Data are frequency (%) or median (interquartile range).

### Clinical Description of ICI-Induced CRS

ICI-induced CRS developed a median of 11.0 days (Q1; Q3 0.0; 24.0) after ICI initiation (**Table 2**). The majority of patients developed mild (Grades 1-2; 17/25; 68.0%) CRS and 8 patients (32.0%) developed severe (Grades 3-5) CRS. Grades 3-4 and Grades 1-2 CRS were similar in proportion amongst patients receiving dual checkpoint blockade (25.0% vs. 11.8%, p=0.400). Patients with Grades 3-5 CRS had longer time to onset of fever onset compared with Grades 1-2 CRS [25.0 days (Q1; Q3 13.0; 136.5) vs. 3.0 days (Q1; Q3 0.0; 18.0), p=0.027]. Time to onset of fever remains statistically significant with a BH FDR of 5% (**Supplemental Table 4**). The median duration of CRS was 15.0 days (Q1; Q3 6.3; 29.8) and 10 (40.0%) had multiple episodes of CRS flares. 13 patients (52.0%) experienced defined non-CRS irAEs, including 3 (12.0%) with Grade 3 or higher irAEs. Among the cases we analyzed, positive microbiological culture (deemed to be a contaminant) was reported in only 1 (4.0%) patient but 20 (80.0%) of them received antibiotics empirically. CRS is clinically defined by a constellation of inflammatory symptoms and signs; CRS-related toxicities in organ systems ranged from cardiovascular (32.0%), dermatological (28.0%), gastrointestinal (36.0%), hepatic (40.0%), neurological (16.0%), pulmonary (16.0%), renal (16.0%) and rheumatic (24.0%) (29). Patients with Grades 3-5 CRS had more cardiovascular (p=0.002), neurologic (p=0.001), pulmonary (p=0.044) and rheumatic (p=0.037) involvement than Grades 1-2 CRS. Severe CRS defining events such as hypotension (p=0.002), use of high dose vasopressors (p=0.007), intubation (p=0.007) and intensive care unit (ICU) admission (p=0.001) were more common in Grades 3-4 compared to Grades 1-2 CRS. Organ systems involvement such as cardiovascular, neurologic, pulmonary and rheumatic, together with hypotension, use of high dose vasopressors, intubation and ICU admission remained statistically associated with severe CRS with a BH adjustment with FDR 10% (**Supplemental Table 4**).

**Table 2:** Clinical factors related to CRS.

Clinical factor	Total cohort (n = 25)	Grade 1-2 (n = 17)	Grade 3-5 (n = 8)	p value*
Fulfill SIRS criteria	25 (100.0)	17 (68.0)	8 (32.0)	N.A.
Age	64.0 (55.0-74.5)	64.0 (56.5-70.0)	66.5 (48.8-81.3)	0.560
Gender (male)	18 (72.0)	11 (64.7)	7 (87.5)	0.236
BMI	22.8 (18.0-25.8)	23.7 (17.7-26.4)	22.0 (19.3-23.6)	0.483
Days to fever	11.0 (0.0-24.0)	3.0 (0.0-18.0)	25.0 (13.0-136.5)	**0.027**
Dual ICI (anti-CTLA-4/anti-PD-1)	4 (16.0)	2 (11.8)	2 (25.0)	0.400
Duration of CRS (days)	15.0 (6.3-29.8)	11.5 (7.3-27.5)	22.0 (2.0-31.5)	0.902
Frequency of CRS episodes				0.861
Single	15 (60.0)	10 (58.8)	5 (62.5)	
Multiple	10 (40.0)	7 (41.2)	3 (37.5)	
Other concomitant irAEs				0.319
No	12 (48.0)	7 (41.2)	5 (62.5)	
Yes	13 (52.0)	10 (58.8)	3 (37.5)	
Grade 3 or more concomitant irAEs	3 (12.0)	2 (11.8)	1 (12.5)	0.837
Positive microbiological cultures	1 (4.0)	1 (5.9)	0 (0.0)	0.484
Antibiotics	20 (80.0)	12 (70.6)	8 (100.0)	0.121
CRS toxicities by organ system				
Cardiovascular	8 (32.0)	2 (11.8)	6 (75.0)	**0.002**
Dermatological	7 (28.0)	6 (35.3)	1 (12.5)	0.236
Gastrointestinal	9 (36.0)	7 (41.2)	2 (25.0)	0.432
Hepatic	10 (40.0)	6 (35.3)	4 (50.0)	0.484
Neurological	4 (16.0)	0 (0.0)	4 (50.0)	**0.001**
Pulmonary	4 (16.0)	1 (5.9)	3 (37.5)	**0.044**
Renal	4 (16.0)	3 (17.6)	1 (12.5)	0.743
Rheumatic	6 (24.0)	2 (11.8)	4 (50.0)	**0.037**
CRS grade-defining events				
Hypotension	8 (32.0)	2 (11.8)	6 (75.0)	**0.002**
High-dose vasopressors used	3 (12.0)	0 (0.0)	3 (37.5)	**0.007**
Oxygen supplementation	20 (80.0)	13 (76.5)	7 (87.5)	0.520
Intubation	3 (12.0)	0 (0.0)	3 (37.5)	**0.007**
ICU admission	4 (16.0)	0 (0.0)	4 (50.0)	**0.001**

*Comparison between Grades 1-2 and 3-5 CRS.

SIRS, systemic inflammatory response syndrome; BMI, body mass index; ICU, intensive care unit. Data are frequency (%) or median (interquartile range). Bold characters in Tables are to emphasize p values < 0.05.

### Laboratory Description of ICI-Induced CRS

We evaluated laboratory markers of inflammation and organ failure at diagnosis and before starting any immunosuppressive treatment, if applicable (**Table 3**). C-reactive protein (CRP) was severely elevated in all patients (median 117.0 mg/L, Q1; Q3 64.0; 195.0). In contrast, procalcitonin was only mildly elevated (median 0.54 µg/L, Q1; Q3 0.32; 1.76). Consistent with generalized inflammation and hypotension, the following parameters were elevated in the majority of patients with CRS: white blood cell count, neutrophil count, NLR, PLR, ferritin (4/25 measured), interleukin (IL)-6 (8/25 measured), alanine aminotransferase (ALT), aspartate aminotransferase (AST), lactate dehydrogenase (LDH), urea, creatinine and lactate (11/25 measured). Patients with Grades 3-5 CRS had lower platelet count [128 (Q1; Q3 101; 424) vs. 298 (Q1; Q3 184; 483), p=0.041] and higher urea [8.4 mmol/L (Q1; Q3 5.6; 13.9) vs. 5.4 mmol/L (Q1; Q3 4.4; 7.5), p=0.041] than Grades 1-2 CRS. Platelet count and urea remained statistically associated with severe CRS with a BH FDR 50% adjustment (**Supplemental Table 5**). CRP (median 67.5 vs. 127.0 mg/L) and IL-6 (median 9.2 vs. 19.3 pg/mL) tended to be lower in Grades 3-5 compared to Grades 1-2 CRS, though this finding was not statistically significant.

**Table 3:** Clinical biomarkers related to CRS*.

Biomarker	At CRS (n = 25)	Grade 1-2 at CRS (n = 17)	Grade 3-5 at CRS (n = 8)	p value**
WBC × 10^9^/L	10.46 (6.75-14.68)	10.46 (6.75-13.31)	10.64 (6.68-20.91)	0.641
Neutrophil × 10^9^/L	7.29 (5.25-13.57)	7.29 (5.35-11.43)	6.96 (5.21-19.81)	0.816
Lymphocyte × 10^9^/L	0.82 (0.52-1.31)	0.82 (0.57-1.32)	0.76 (0.46-1.29)	0.705
Platelet × 10^9^/L	245 (129-464)	298 (184-483)	128 (101-424)	**0.041**
NLR	7.36 (4.27-25.50)	7.35 (3.92-19.39)	11.46 (4.54-38.31)	0.449
PLR	298.78 (173.40-532.35)	316.92 (249.45-580.59)	164.89 (104.58-557.25)	0.145
Hemoglobin (g/dL)	9.4 (8.1-11.1)	9.6 (8.4-11.1)	8.8 (7.9-10.9)	0.431
CRP (mg/L)	117.0 (64.0-195.0)	127.0 (66.0-213.5)	67.5 (63.5-150.8)	0.180
Procalcitonin (µg/L)	0.54 (0.32-1.76)	0.47 (0.30-1.15)	1.31 (0.34-11.71)	0.241
Ferritin (µg/L)	1078 (720-1398)	1251 (1078-)	776 (664-)	0.248
IL-6 (pg/mL)	10.1 (5.0-28.4)	19.3 (3.8-50.5)	9.2 (4.8-15.2)	0.462
ALT (U/L)	34 (17-109)	26 (17-137)	37 (10-106)	0.923
AST (U/L)	37 (29-145)	36 (26-116)	50 (30-295)	0.438
LDH (U/L)	690 (447-1211)	603 (431-1037)	967 (460-4601)	0.245
Urea (mmol/L)	6.3 (4.6-8.2)	5.4 (4.4-7.5)	8.4 (5.6-13.9)	**0.041**
Creatinine (µmol/L)	65 (59-100)	61 (59-93)	74 (58-315)	0.414
Lactate (mmol/L)	2.5 (1.3-6.7)	1.8 (1.1-3.2)	5.8 (2.1-11.0)	0.068

*Data extracted before starting an immunosuppressive treatment

**Comparison between Grades 1-2 and 3-5 CRS.

WBC, white blood cell; NLR, neutrophil-to-lymphocyte ratio; PLR, platelet-to-lymphocyte ratio; CRP, C-reactive protein; ALT, alanine aminotransferase; AST, aspartate aminotransferase; LDH, lactate dehydrogenase. *Data are or median (interquartile range). Bold characters in Tables are to emphasize p values < 0.05.

### Treatment of ICI-Induced CRS and Impact of Tumor Response

Overall, immunosuppressive treatment was required in 18 patients (72.0%) to treat the ICI-induced CRS (**Table 4**). 9 patients (36.0%) were treated with pulse methylprednisolone, 1 patient (4.0%) was treated with dexamethasone for neurologic toxicity and 6 patients (24.0%) were treated with tocilizumab. Use of pulse methylprednisolone (p=0.045) and tocilizumab (p=0.016) varied significantly by grades of CRS but cumulative prednisolone dose did not differ significantly. Use of methylprednisolone and tocilizumab remained statistically significant with a BH FDR 15% adjustment (**Supplemental Table 6**). More patients with Grade 1 CRS responded to steroids with resolution of fever compared to Grade 5 CRS [4 (66.7%) vs. 1 (16.7%), p=0.016]. 7 patients (28.0%) with Grades 1-2 CRS were rechallenged using ICIs without event. In this limited sample size, tumor response did not differ significantly amongst patients experiencing different grades of CRS.

**Table 4:** Treatment and outcome of CRS.

	Total cohort (n = 25)	Grade 1 (n =6)	Grade 2 (n = 11)	Grade 3 (n = 1)	Grade 4 (n = 1)	Grade 5 (n = 6)	p value*
Tocilizumab	6 (24.0)	1 (16.7)	0 (0.0)	1 (100.0)	1 (100.0)	3 (50.0)	**0.016**
Single dose		1 (16.7)			1 (100.0)	3 (50.0)	
Multiple doses				1 (100.0)			
Pulse methylprednisolone	9 (36.0)	0 (0.0)	3 (27.3)	1 (100.0)	1 (100.0)	4 (66.7)	**0.045**
Cumulative prednisolone dose (mg)	632.5 (228.8-1866.3)	273.8 (18.8-1919.6)	700.0 (255.0-1085.0)	1809.2 (1809.2-1809.2)	8535.0 (8535.0-8535.0)	565.0 (325.0-1590.0)	0.398
Steroid response	12 (48.0)	4 (66.7)	6 (54.5)	1 (100.0)	1 (100.0)	1 (16.7)	0.058
ICI rechallenged	7 (28.0)	4 (66.7)	3 (27.3)	0 (0.0)	0 (0.0)	0 (0.0)	0.054
RECIST**							0.251
Partial response		2 (33.3)	3 (27.3)		1 (100.0)		
Stable disease			3 (27.3)	1 (100.0)		1 (16.7)	
Progressive disease		4 (66.7)	3 (27.3)			3 (50.0)	

* Comparison between Grades 1, 2, 3, 4 and 5 CRS.

** Missing data were not evaluable.

RECIST, Response Evaluation Criteria In Solid Tumors. Data are frequency (%) or median (interquartile range). Bold characters in Tables are to emphasize p values < 0.05.

### Cytokine Release Syndrome After RT

**Figure 1:**
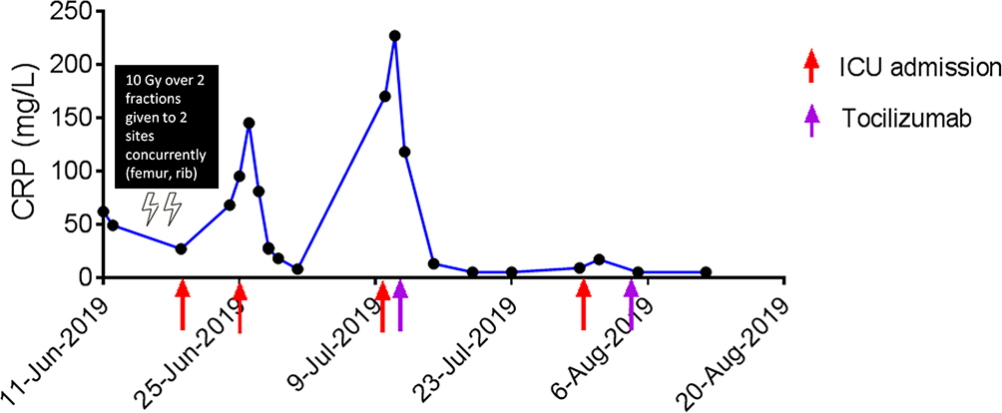
Response to tocilizumab in a patient with CRS after RT.

One patient had recurrent CRS flares requiring multiple doses of tocilizumab (8 mg/kg). A 59-year-old Chinese male started on ICIs for treatment of clear cell renal cell carcinoma metastatic to lung, bone and lymph nodes. He received 4 cycles of ipilimumab and nivolumab followed by 4 cycles of maintenance nivolumab. The metastases progressed during immunotherapy and the patient was planned for a course of palliative RT (25 Gy given over 5 fractions) to the symptomatic soft tissue deposits in the femur and left 2^nd^ rib. Two fractions of RT (i.e., total 10 Gy) were administered to both sites concurrently (over 2 consecutive days). Prior to the 3^rd^ fraction, the patient developed CRS at 195 days after the start of immunotherapy and 37 days after the last dose of nivolumab, and was admitted to the ICU 4 times for hypotension and desaturation (**Figure 1**). This patient received pulse methylprednisolone followed by oral prednisolone but had recurrence of CRS whenever prednisolone was stopped or tapered to a lower dose (7.5 mg/day). The patient received 2 cycles of tocilizumab (**Figure 1**) with good effect. He received another 2 more cycles of maintenance tocilizumab outpatient before passing away from intracranial hemorrhage due to brain metastasis.

## Discussion

Cytokine release syndrome is among the most serious adverse events and has been described in various terms in both oncology and non-oncology literature. In addition to CAR-T and BiTE therapy, ICI immunotherapy is now a recognized cause CRS (15). The prevalence of ICI-induced CRS is 4.6% or 4,638 per 100,000 based on our study. This is more common than currently being reported, and well above that of rarer organ-specific irAEs (4). However, ICI-induced CRS is still not included in major practice guidelines and it does not have any diagnostic criteria or treatment guidelines (25–28). Despite the relative frequency of ICI-induced CRS, little is known about the biology of this syndrome, and much is still extrapolated from the CAR-T and BiTE therapy literature. CRS is usually due to on-target on-tumor effects induced by binding of the CAR-T cell receptor or bispecific antibody to its antigen and subsequent activation of myeloid cells (e.g. monocyte lineage cells and macrophages) and non-immune cells (e.g. endothelial cells) (12, 20, 23, 31). In addition, the strength of T cell activation and degree of T cell expansion correlate with the severity of CRS (20). Interferon (IFN)-γ, IL-6, IL-8, IL-10 and tumor necrosis factor (TNF)-α are among the core cytokines that are found elevated in the serum of patients with CRS (20, 31). CRS is triggered by the massive release of IFN-γ by activated T cells or tumor cells, which in turn induces activation of macrophages (20). The activated macrophages produce excessive amounts of IL-6, IL-8, IL-10 and TNF-α (24). Levels of IL-1β, its natural antagonist IL-1 receptor antagonist (IL-1Ra) and granulocyte-macrophage colony-stimulating factor (GM-CSF) tend to peak earlier than the above cytokines, suggesting that IL-1β, IL-1Ra and GM-CSF might have roles in initiating the inflammatory cascade (31). Generally, cytokines that are differentially elevated based on CRS grades include cytokines released from activated T cells or activated monocyte/macrophages, chemokines that are chemotactic for monocytes/macrophages and cytokines that are often elevated after tissue damage and inflammation (24). Of note, IFN-γ is not expected to be elevated in sepsis and may help to differentiate ICI-induced CRS from patients with infection (20, 24).

Cytokine release syndrome typically presents with fever and temperatures frequently exceed 40°C, meeting criteria for SIRS (13, 29). More severe cases are characterized by hypotension and capillary leak, often leading to hypoxia and pulmonary edema (11). In keeping with this, we observed that patients with severe grades of ICI-induced CRS had more hypotension, use of high dose vasopressors, intubation and ICU admission in our study. CRS can also affect many different organ systems and cause dysfunction (29, 31). Organ dysfunction may occur secondary to hypotension or hypoxia but may also result from direct effect of cytokine release (31). Of note, neurologic toxicity, also known as immune effector cell-associated neurotoxicity syndrome, is due to endothelial cell activation and disruption of the blood-brain barrier (BBB), with accumulation of cytokines and T cells in the central nervous system (CNS) together with activation of microglial cells (31). We found that patients with severe grades of ICI-induced CRS had more neurologic and rheumatic involvement in our series. Rheumatic toxicity refers to myalgia or weakness with elevated muscle enzymes but the pathophysiology is not well defined (29). In severe cases, CRS can be accompanied by features of macrophage activation syndrome (MAS), characterized by hyperferritinaemia, prolonged cytopenias, coagulopathy and liver function abnormalities (20, 31). CRS and MAS are overlapping conditions, and MAS should be considered as a complication of ICI-induced CRS (32). In patients with CRS who develop MAS, additional cytokines like IL-8, IL-18, IFN-induced protein 10, monocyte chemoattractant protein-1, monokine induced by IFN-γ and macrophage inflammatory protein-1β are also elevated (20). Ferritinaemia greater than 10,000 µg/L has a sensitivity of 90% and specificity of 96% for MAS (33). We did not observe any patient with MAS in our case series; the ferritin was only mildly elevated (median 1078 µg/L, Q1; Q3 720; 1398). Why some patients develop severe CRS is poorly understood and may be due to genetic variants predisposing them (20). Genetic variants in *Il6* gene may lead to overexpression of IL-6 and it has been suggested that polymorphisms in the *Il6* gene could predispose patients to ICI-induced CRS (15).

Interleukin-6 holds a key role in CRS immunopathogenesis since highly elevated IL-6 levels are seen in patients with CRS (20). IL-6, a pleiotropic cytokine with broad ranging biological effects on both immune and non-immune cells, is often targeted for the management of irAEs (34). Observational studies have demonstrated clinical improvement in patients with a broad range of irAEs after receiving the anti-IL-6-receptor (IL-6R) mAb, tocilizumab (13, 34). IL-6R is found on macrophages, neutrophils, hepatocytes and some T cells, and mediates classical signaling which predominates when IL-6 levels are low. However, when IL-6 levels are elevated, soluble IL-6R can also initiate trans-signaling on a much wider variety of cells (23). Current models hold that anti-inflammatory effects of IL-6 are mediated by classical signaling, whereas proinflammatory responses are mediated by trans-signaling (23). *Via* trans-signaling, IL-6 leads to characteristic manifestations of severe CRS, e.g. vascular leakage, activation of complement and coagulation cascade inducing disseminated intravascular coagulation (20). Single-cell RNA-sequencing data of leukocytes isolated during CRS have also confirmed that monocyte lineage cells are the main source of IL-6 (31). The current management approach of CRS is to administer tocilizumab to all patients experiencing ≥ Grade 3 CRS, and to patients with ≥ Grade 2 CRS with comorbidities or elderly (23). However, severe neurologic toxicities are treated with systemic corticosteroids rather than tocilizumab due to concerns for the mAb to cross the BBB. Dexamethasone is often chosen for in this context because of its excellent CNS penetration (29). In patients who respond to tocilizumab, fever and hypotension often resolve within a few hours, vasopressors and other supportive measures can be weaned quickly after (23). Stroud et al. described 12 patients with Grades 3-4 CRS following nivolumab and were treated with tocilizumab (13). However, their responses to tocilizumab were not reported (13). In our case series, 6 patients (24.0%) were treated with tocilizumab and 1 patient (4.0%) was treated with dexamethasone for concomitant neurologic toxicity. Despite this, 3 patients (50%) who received tocilizumab had fatal (Grade 5) outcomes from ICI-induced CRS. The goal of these treatments is to avoid life-threatening toxicity (29). However, predictors of severe ICI-induced CRS have not been reported in the literature for earlier initiation of treatment with tocilizumab.

C-reactive protein is an acute phase reactant that is produced by the liver largely in response to IL-6, and CRP levels serve as a reliable surrogate for IL-6 bioactivity (23). CRP has been suggested as a biomarker for determining severity of CRS (20). Elevation of CRP ≥ 200 mg/L correlates with severe CRS with a specificity of 100% (29). However, CRP by itself is insufficient to predict the severity of CRS (20). A CRP-based grading scale for CRS, using a combination of clinical features, cytokines and CRP has been proposed (11). However, IL-6 and CRP were paradoxically lower in Grades 3-5 CRS compared to Grades 1-2 ICI-induced CRS in our study. The levels of IL-6 are relatively low in this study. This could be due to treatment with immunosuppression for the ongoing CRS as the IL-6 would have been ordered after the rheumatology consult. Disease burden at time of treatment has been associated with severity of CRS but similarly, disease burden alone is not sufficient as a predictor of severe CRS (24). We found that longer time to fever onset, lower platelet count and higher urea at presentation were associated with Grade 3-5 ICI-induced CRS. These constellation of clinical findings may reflect the following: (i) prevention of dysfunction of newly recruited tumor-specific T cells from the periphery rather than reinvigoration of dysfunctional lymphocytes already present in the tumor, thus leading to a slower and more robust immune response; (ii) lower platelet count due to macrophage activation; (iii) renal dysfunction due to hypotension and (iv) possibly due to circular reasoning as a result of categorizing patients with Grade 3-4 renal toxicity under the category of severe CRS, respectively (23, 35). Other factors, such as RT or vaccination with vaccines (e.g. BTN162b2 – the Pfizer-BioNTech mRNA coronavirus 2019 vaccine) have been newly reported to trigger CRS events in patients receiving ICIs (36, 37). The close temporal association of RT and onset of CRS in our case described above also favors RT as a cofactor for triggering CRS. The contribution of the RT dose, fraction size and site of radiation, to this phenomenon, remains unclear. In the previously reported case, the patient received 24 Gy over 3 fractions (i.e. 8 Gy per fraction) to the inguinal and gluteal regions (36). Co-medication with PPIs has been reported with occurrence of renal irAEs and coincidentally, most of the patients in this study were on PPIs (38).

The current study has several limitations and strengths. First, our study is mainly limited by its retrospective nature and relatively small sample size. To address the issue of multiple comparisons in a relatively small sample size, BH corrections were performed to test the statistical significance of results. Second, our study population was obtained from 2 tertiary hospitals in Singapore, which might limit generalizing the results to other populations or ethnicities. Future larger multi-center prospective studies would be useful in validating our findings. Third, we did not analyze other cytokines, chemokines and soluble receptors. On this note, personalized treatment for organ-specific irAEs have been proposed based on the immunopathological analyses and the closest analogy for ICI-induced CRS would be a bone marrow aspirate for concomitant MAS (32, 34). We recommend that peripheral blood flow cytometery and measurement of peripheral blood cytokines (e.g. IL-1β, IL-6 and TNF-α) should be performed whenever feasible to further refine the therapeutic decision-making for CRS (34). Lastly, milder grades of CRS are hard to distinguish from other causes of SIRS and we do recognize some patients may have been incorrectly been classified as having CRS. Nevertheless, this study also has considerable strengths. This is the largest case series, to our knowledge, to analyze the clinical characteristics of these patients, factors and biomarkers associated with high grade ICI-induced CRS, and their outcomes in detail. In addition, our data were derived from multi-ethnic patients with different cancer types receiving various classes of ICIs, reflecting a real-world scenario.

## Conclusions

Immune checkpoint inhibitors are used with increasing frequency and represent standard of care in some cancers. Knowledge about irAEs is essential for effective clinical management of patients receiving ICIs. CRS represents an increasingly important irAE, and early diagnosis and timely intervention are required for good outcomes. CRS should be in the differential diagnoses of any patient treated with ICIs presenting with SIRS. Longer time to fever onset, lower platelet count and higher urea at presentation are associated with Grade 3-5 ICI-induced CRS and may be used to predict which patients are likely to develop severe CRS before they become critically ill. Rheumatologists and allergists-immunologists may be best equipped to manage ICI-induced CRS since they are the clinicians most closely and historically associated to immunology and the management of systemic inflammatory diseases (39). Overall, close collaboration between rheumatologists, allergists-immunologists and medical oncologists is required to achieve a better understanding of the immunopathogenesis and determine the best clinical care for patients with ICI-induced CRS.

## Data Availability Statement

The datasets presented in this article are not readily available because data are not publicly available due to privacy and ethical restrictions. Requests to access the datasets should be directed to sen_hee_tay@nuhs.edu.sg.

## Ethics Statement

The studies involving human participants were reviewed and approved by NHG Domain Specific Review Board B. The patients/participants provided their written informed consent to participate in this study.

## Author Contributions

SHT performed literature review; MMXT and YLT collected and curated the data; SHT, ASCW and YHC performed data analysis and interpretation; AM, BAV, A-MF, FA, LDB, YH and ASCW participated in discussions; SHT drafted the manuscript; and all authors critically reviewed and approved the final version of the manuscript.

## Funding

This work was supported by National Research Foundation, Singapore and National Medical Research Council, Singapore under its NMRC Centre Grant Programme (NMRC/CG/M005/2017_NCIS).

## Conflict of Interest

The authors declare that the research was conducted in the absence of any commercial or financial relationships that could be construed as a potential conflict of interest.

## Publisher’s Note

All claims expressed in this article are solely those of the authors and do not necessarily represent those of their affiliated organizations, or those of the publisher, the editors and the reviewers. Any product that may be evaluated in this article, or claim that may be made by its manufacturer, is not guaranteed or endorsed by the publisher.
