# Supplementary material for: Cytokine Release Syndrome in Cancer Patients Receiving Immune Checkpoint Inhibitors: A Case Series of 25 Patients and Review of the Literature
Source: Front Immunol. 2022 Jan 28;13:807050. doi: 10.3389/fimmu.2022.807050 (PMC8831742; doi:10.3389/fimmu.2022.807050)
Supplement: Supplementary file 1 [file DataSheet_1.zip › Supplementary Material.docx]

Supplementary Material

**Supplemental Table 1.** Attribution of SIRS events to CRS.

Attribution Mode A (more stringent): SIRS events attributed to CRS had no “exclusion”.

OR

Attribution Mode B (less stringent): SIRS events attributed to CRS had “associations”.

| **Exclusion** | **Associations** |
| --- | --- |
| Positive microbiological culture within 1 week of inpatient investigations for pyrexia | Positive microbiological culture within 1 week of inpatient investigations for pyrexia    AND  Infection disease physician deemed CRS accounted for presenting SIRS event despite infection or positive microbiological culture thought to be contaminant |
|  |  |

**Supplemental Table 2.** Characteristics of 25 patients with ICI-induced CRS.

| **Patient No.** | **Age/**  **Gender** | **Tumor/Stage** | **Prior treatment** | **ICI** | **Time to CRS (Days)/**  **Duration of ICI (Days)** | **Grade of CRS** | **Manifestations of CRS** | **Laboratory parameters** | **Treatment of CRS/**  **Response to CRS treatment** | **RECIST** |
| --- | --- | --- | --- | --- | --- | --- | --- | --- | --- | --- |
| 1 | 44/Male | Adrenocortical carcinoma/IV | EDP-M | Nivo | 0/63 | 1 | Fever | WBC 6.71  PLT 156  CRP 200 | Supportive care | Progressive disease |
| 2 | 70/Female | Melanoma/IV | Adjuvant RT | Nivo | 0/107 | 1 | Fever and transaminitis | WBC 6.79  PLT 328  CRP 117  IL-6 19.3 | Prednisolone 30 mg/day and tapered to 5 mg/day, with resolution of fever 1 day after starting prednisolone  Nivolumab continued and had flare of CRS when prednisolone stopped  IV tocilizumab 8 mg/kg × 1 | Progressive disease |
| 3 | 64/Female | NSCLC/IV | Carboplatin/  pemetrexed | Nivo | 23/28 | 1 | Fever, lethargy dyspnea and transaminitis | WBC 41.51  PLT 535  CRP 214 | Started on prednisolone 40 mg/day (1 mg/kg/day) with persistent fevers.  Prednisolone increased to 60 mg/day 3 days later with immediate response, tapered and withdrawn | Progressive disease |
| 4 | 64/Male | Renal cell carcinoma/IV | Nil | Nivo | 19/275 | 1 | Fever | WBC 9.54  PLT 612  CRP 182 | Supportive care | Partial response |
| 5 | 61/Female | Melanoma/IV | Nil | Pembro,  Nivo + Ipi | 173/231 | 1 | Fever | WBC 9.01  PLT 333  CRP 83  IL-6 63.4 | Supportive care  Prednisolone 5 mg/day started because of ICI-associated adrenal insufficiency | Progressive disease |
| 6 | 75/Male | NSCLC/IV | Nil | Nivo | 0/543 | 1 | Fever | WBC 19.74  PLT 566  CRP 213 | Supportive care | N.A. |
| 7 | 63/Male | Renal cell carcinoma/IV | RT | Nivo + Ipi | 0/21 | 2 | Fever and transaminitis  Liver biopsy done compatible with drug-induced liver injury | WBC 12.72  PLT 412  ALT 543  AST 518  CRP 17 | IV methyl-prednisolone 2 mg/kg/day × 3 followed by prednisolone 60 mg/day  Myfortic 720 mg BD  Marked resolution of fever, gradual improvement in transaminitis | Stable disease |
| 8 | 57/Male | NSCLC/IV | Carboplatin/ pemetrexed | Pembro | 3/497 | 2 | Fever with new onset atrial flutter and dermatitis | WBC 13.87  PLT 180  CRP 265 | Prednisolone 30 mg/day, tapered by 5 mg every 3 days and withdrawn  Responded well with only 1 episode of low grade temperature while on prednisolone 25 mg/day | Partial response |
|  | 67/Female | Endometrial carcinoma/IV | Adjuvant carboplatin/ paclitaxel/ RT  Carboplatin/ paclitaxel/ bevacizumab | Pembro | 3/21 | 2 | Fever | TW 12.75  PLT 298  CRP 214 | Supportive care | Progressive disease |
| 10 | 70/Male | NSCLC/IV | Nil | Pembro | 1/21 | 2 | Fever and hypotension  BP 83/53 mmHg | WBC 11.6  PLT 285  CRP 170  IL-6 6.3  Lactate 1.8 | Hypotension responsive to fluids  I IV methyl-prednisolone 2 mg/kg/day × 3 followed by prednisolone 60 mg/day  Fever and hypotension resolved promptly after starting IV methyl-prednisolone | Progressive disease |
| 11 | 56/Male | NSCLC/IV | Nil | Pembro | 0/21 | 2 | Fever, vomiting and diarrhea | WBC9.87  PLT 892  CRP 343 | IV methyl-prednisolone 2 mg/kg/day × 3 followed by prednisolone 60 mg/day for treatment of ICI-induced enteritis  Fever, vomiting and diarrhea resolved promptly after starting IV methyl-prednisolone | N.A. |
| 12 | 46/Male | Renal cell carcinoma/IV | Axitinib and RT | Nivo | 0/123 | 2 | Fever, hypotension and acute renal impairment  BP 80/39 mmHg  Developed later SJS/TEN after being started on prednisolone, attributed to nivo | WBC 10.46  PLT431  CRP 40  Lactate 2.5 | Prednisolone 40 mg/day and then increased to 50 mg/day for SJS/TEN  Fever and hypotension resolved promptly after starting prednisolone | Stable disease |
| 13 | 76/Male | Hepatocellular carcinoma | Bevacizumab | Durv | N.A. | 2 | Fever, myositis, dermatitis and renal impairment  CRS occurred when prednisolone tapered from 30 mg/day to 20 mg for treatment of dermatitis | WBC 11.06  PLT 129  Cr 142  CRP 127 | Prednisolone 30 mg/day  Information on CRS treatment response not available as patient was transferred to another hospital |  |
| 14 | 78/Female | Hepatocellular carcinoma/IV | Levantinib | Pembro | 2/90 | 2 | Fever, dermatitis, cytopenias and transaminitis | WBC 3.38  PLT 129  ALT 141  AST 238  CRP 70  Ferritin 1424  IL-6 37.5 | Supportive care | Partial response |
| 15 | 58/Male | Esophageal SCC/IV | FOLFOX | Pembro | 36/78 | 2 | Fever, hypotension and transaminitis  BP 75/49 mmHg | WBC 15.48  PLT 223  ALT 193  AST 233  CRP 109 | IV hydrocortisone 100 mg q 8H and converted to prednisolone 35 mg/day, tapered and withdrawn  Marked resolution of fever and hypotension, gradual improvement in transaminitis | Partial response |
| 16 | 70/Male | NSCLC/IV | Carboplatin/ paclitaxel | Pembro | 12/157 | 2 | Fever, hypotension, diarrhea and dermatitis | WBC 3.05  PLT 188  CRP 45  Ferritin 1078  IL-6 1.3  IFNγ 16.9 | IV hydrocortisone 100 mg q 8H and converted to prednisolone 50 mg/day, tapered and withdrawn  Fever and hypotension resolved promptly after starting hydrocortisone | N.A. |
| 17 | 44/Female | Breast cancer/IV | Carboplatin/ paclitaxel | Anti-PD1 and anti-LAG3 | 3/28 | 2 | Fever, diarrhea and dermatitis | WBC 3.45  PLT 245  CRP 62 | Supportive care | N.A. |
| 18 | 59/Male | Renal cell carcinoma/IV | Nil | Nivo + Ipi, followed by RT | 195/141 | 3 | Recurrent episodes of unexplained fevers, hypotension and respiratory failure  Admitted to ICU 4 times but did not require intubation | WBC 10.3  PLT 543  CRP 68 | IV methyl-prednisolone 2 mg/kg/day converted to prednisolone 45 mg/day and tapered  PO dexamethsone for brain metastases  IV tocilizumab 8 mg/kg × 4  Details of response to CRS treatment in main text and Figure 1 | Progressive disease |
| 19 | 44/Male | Hepatocellular carcinoma/IV |  | Atezo | 1/11 | 4 | Fever, hypotension respiratory requiring ICU admission and intubation | WBC 4.3  PLT 119  CRP 10.3  Lactate 10.9  IL-6 10.1 | IV methyl-prednisolone and IV tocilizumab 8 mg/kg  Fever and hypotension resolved after starting tocilizumab | Partial response |
| 20 | 84/Male | Transitional cell carcinoma/IV | Gemcitabine/ paclitaxel  RT | Pembro | 15/31 | 5 | Fever and hypotension requiring IV dopamine | WBC 10.98  PLT 95  CRP158  Lactate 2.4 | IV methyl-prednisolone 2 mg/kg/day × 15  IV tocilizumab 4 mg/kg/day × 1  No response to IV methyl-prednisolone and IV tocilizumab | Progressive disease |
| 21 | 54/Male | NSCLC/IV | Nil | Ave | 0/2 | 5 | New onset of respiratory failure and hypotension during infusion of 1^st^ dose of Ave  Admitted for ICU for intubation, noradrenaline and renal impairment on dialysis | TW 21.45  PLT ×496  CRP 129  Lactate 11.2 | IV methyl-prednisolone 2 mg/kg/day  IV tocilizumab 4 mg/kg/day × 1  No response to IV methyl-prednisolone and IV tocilizumab | N.A. |
| 22 | 74/Male | Hepatocellular carcinoma/IV | FOLFOX | Nivo | 39/56 | 5 | Fever and hypotension requiring ICU admission  Required IV dopamine infusion | WBC 5.3  PLT 129  CRP 65  IL-6 3.6 | IV methyl-prednisolone 2 mg/kg/day × 6  IV tocilizumab 4 mg/kg/day × 1  No response to IV methyl-prednisolone and IV tocilizumab | Stable disease |
| 23 | 79/Female | Colorectal cancer/IV | FOLFOX and cetuximab | Pembro | 78/87 | 5 | Fever, fatigue and delirum attributed to CRS-related encephalopathy | WBC 42.33  PLT 126  CRP 190  IL-6 16.9 | IV methyl-prednisolone 2 mg/kg/day × 4 and converted to prednisolone 50 mg/day  Resolution of fever and delirium after starting IV methyl-prednisolone and converted to prednisolone  Developed acute onset type 1 respiratory failure and death while on prednisolone (5 days after starting CRS treatment) | Progressive disease |
| 24 | 47/Male | NPC/IV | Gemcitabine/ cisplatin | Nivo + Ipi | 25/26 | 5 | Fever, confusion, hypotension, transaminitis with resultant liver failure  BP 74/46 mmHg | WBC 19.28  PLT 47  ALT 109  AST 295  CRP 67 | Supportive care | Progressive disease |
| 25 | 82/Male | NSCLC/IV | Carboplatin/ paclitaxel  RT | Nivo | 25/31 | 5 | Fever, arthralgia and delirium attributed to CRS-related encephalopathy | WBC 6.23  PLT 206  CRP 63  IL-6 8.3 | IV dexamethasone 10 mg q 6 H × 8 doses  No response to IV dexamethasone | N.A. |

*Abbreviations: NSCLC, non-small cell lung cancer; NPC, nasopharyngeal carcinoma; SCC, squamous cell carcinoma; EDP-M, Etoposide, doxorubicin and cisplatin plus mitotane; FOLFOX, 5-fluorouracil and oxaliplatin; RT, radiotherapy; Nivo, nivolumab; Pembro, pembrolizumab; Durv, durvalumab; Ipilimumab, ipi; Atezo, atezolimumab; Ave, avelumab; SJS/TEN, Stevens-Johnson syndrome/toxic epidermal necrolysis; ICU, intensive care unit; WBC, white blood cell; PLT, platelet; CRP, C-reactive protein; IL-6, interleukin 6; ALT, alanine aminotransferase; AST, aspartate aminotransferase; Cr, creatinine

**Units: WBC (10^9^/L); PLT (10^9^/L); CRP (mg/L); IL-6 (pg/mL); ALT (U/L); AST (U/L); ferritin (μg/L); lactate (mmol/L)

**Supplementary Table 3.** Baseline characteristics of the National University Hospital ICI patient cohort.

| **Demographics** | **n = 485 (%)** |
| --- | --- |
| **Gender**  Male  Female | 321 (66.2%)  164 (33.8%) |
| **Age, median** | 61 |
| **ECOG**  0 - 1  2  3 – 4  Unknown | 310 (63.9%)  27 (5.6%)  10 (2.1%)  138 (28.4%) |
| **Race**  Chinese  Malay  Indian  Others | 367 (75.7%)  50 (10.3%)  25 (5.2%)  43 (8.9%) |
| **Smoking**  Yes  No | 269 (55.5%)  216 (44.5%) |
| **Type of ICI**  Pembrolizumab  Nivolumab  Atezolizumab  Durvalumab  Tremelimumab  Ipilimumab  Others | 239 (48.6%)  121 (24.6%)  57 (11.6%)  56 (11.4%)  4 (0.8%)  1 (0.2%)  13 (2.6%) |
| **Line of Treatment**  1^st^  2^nd^  3^rd^  4th and beyond | 207 (42.7%)  139 (28.7%)  69 (14.2%)  70 (14.4%) |
| **Duration of treatment (days) median** | 131 |
| **Type of cancer** |  |
| Lung | 238 (49.1%) |
| Renal | 52 (10.7%) |
| Liver | 28 (5.4%) |
| Breast  Colorectal | 26 (5.1%)  25 (5.1%) |
| Nasopharynx | 22 (4.5%) |
| Stomach  Esophageal | 19 (3.9%)  16 (3.3%) |
| Head and Neck  Melanoma | 14 (2.8%)  11 (2.2%) |
| Urothelial | 8 (1.6%) |
| Ovary  Cervix  Lymphoma  Others | 6 (1.2%)  6 (1.2%)  4 (0.7%)  10 (2.0%) |

* Abbreviations: ICI, Immune checkpoint inhibitors; ECOG, Eastern Cooperative Oncology Group

**Supplemental Table 4.** Application of BH procedure for all variables in Table 2 to control for FDRs. Variables that were originally statistically significant in Table 2 are displayed.

|  |  | False Discovery Rate | |
| --- | --- | --- | --- |
|  | Original p values from Table 2 | 5% | 10% |
|  |  | Critical values | |
| Days to fever | 0.027 | 0.0152 | 0.0304 |
| Cardiovascular | 0.002 | 0.0065 | 0.0130 |
| Neurological | 0.001 | 0.0022 | 0.0043 |
| Pulmonary | 0.044 | 0.0196 | 0.0391 |
| Rheumatic | 0.037 | 0.0174 | 0.0348 |
| Hypotension | 0.002 | 0.0087 | 0.0174 |
| High-dose vasopressors used | 0.007 | 0.0109 | 0.0217 |
| Intubation | 0.007 | 0.0130 | 0.0261 |
| ICU admission | 0.001 | 0.0043 | 0.0087 |

*Variable remains significant if original p value from table are less than critical values

** Number of comparisons = 23

**Supplemental Table 5.** Application of BH procedure for all variables in Table 3 to control for FDRs. Variables that were originally statistically significant in Table 3 are displayed.

|  | Original p value from  Table 3 | False Discovery Rate |
| --- | --- | --- |
|  |  | 50% |
|  |  | Critical values |
| Platelet × 10^9^/L | 0.041 | 0.044 |
| Urea (mmol/L) | 0.041 | 0.044 |

*Variable remains significant if original p values from tables are less than critical values

** Number of comparisons = 17

**Supplemental Table 6.** Application of BH procedure for all variables in Table 4 to control for FDRs. Variables that were originally statistically significant in Table 4 are displayed.

|  | Original p value from Table 4 | False Discovery Rate |
| --- | --- | --- |
|  |  | 15% |
|  |  | Critical values |
| Tocilizumab | 0.016 | 0.025 |
| Pulse methylprednisolone | 0.045 | 0.05 |

*Variable remains significant if original p values from table are less than critical values

** Number of comparisons = 6
